# Total internal reflection fluorescence anisotropy imaging microscopy: setup, calibration, and data processing for protein polymerization measurements in living cells

**DOI:** 10.1088/2050-6120/aa872e

**Published:** 2017-12-19

**Authors:** Florian Ströhl, Hovy H W Wong, Christine E Holt, Clemens F Kaminski

**Affiliations:** 1 Department of Chemical Engineering and Biotechnology, University of Cambridge, Philippa Fawcett Drive, Cambridge, CB3 0AS, United Kingdom; 2 Department of Physiology, Development and Neuroscience, University of Cambridge, Downing Street, Cambridge, CB2 3DY, United Kingdom; fs417@cam.ac.uk

**Keywords:** anisotropy, polymerization, TIRF, live cell

## Abstract

Fluorescence anisotropy imaging microscopy (FAIM) measures the depolarization properties of fluorophores to deduce molecular changes in their environment. For successful FAIM, several design principles have to be considered and a thorough system-specific calibration protocol is paramount. One important calibration parameter is the *G* factor, which describes the system-induced errors for different polarization states of light. The determination and calibration of the *G* factor is discussed in detail in this article. We present a novel measurement strategy, which is particularly suitable for FAIM with high numerical aperture objectives operating in TIRF illumination mode. The method makes use of evanescent fields that excite the sample with a polarization direction perpendicular to the image plane. Furthermore, we have developed an ImageJ/Fiji plugin, AniCalc, for FAIM data processing. We demonstrate the capabilities of our TIRF-FAIM system by measuring $\beta $-actin polymerization in human embryonic kidney cells and in retinal neurons.

## 1. Introduction

Fluorescence anisotropy imaging microscopy (FAIM) is used to spatially resolve and quantify a range of physical and chemical properties, including rotational diffusion (RD) [1], polymerization reactions [2], and conformational changes of a molecule [3]. These properties can be measured via the fluorescence anisotropy *r*, which is defined as

1 \begin{eqnarray*}r=\displaystyle \frac{{I}_{\parallel }-{{GI}}_{\perp }}{{I}_{\parallel }+2{{GI}}_{\perp }}.\end{eqnarray*}

Here, ${I}_{\parallel }$ and ${I}_{\perp }$ are the measured fluorescence intensities filtered by analyzers with their transmission axis aligned either in parallel with, or perpendicular to, the polarization direction of the excitation light. For the accurate measurement of the extent of depolarization of fluorescent molecules, one needs to know the systematic errors introduced by the optical set-up and the detectors in use, which are combined in a single parameter commonly referred to as the *G* factor. This name stems from polarization-sensitive spectrofluorometers, which use gratings, hence ‘*G* factor’, for spectral selection. Grating efficiency is highly polarization dependent, hence its influence on the signal recorded by, for example, a photodiode must be properly accounted for. In equation (1), the factor 2 in the denominator ensures that the polarized components are normalized by the total intensity: in three-dimensions there exist two perpendicular components to the excitation light polarization, one in the image plane and one along the optical axis, which have the same amplitude. In FAIM imaging spectral filters are used for wavelength selection, which exhibit a smaller polarization dependence ($G\approx 1$) than gratings. Usually the signals for ${I}_{\parallel }$ and ${I}_{\perp }$ are detected by different pixel elements. Often two distinct cameras are used for their detection or the respective signals are used on separate parts of the same camera chip using an image splitter. This means that the relative sensitivity of the pixels need to be taken into account via the *G* factor. Also, as multiple pixels record the signal, the *G* factor becomes a matrix, with each entry representing a pixel-pair between two recording cameras. Note that the *G* factor is a system parameter and, although independent of illumination power and sample concentration, it is dependent on wavelength. Three different methods are commonly employed to determine the *G* factor. In a spectrofluorometer, where illumination and emission paths are at right angles (called L-format), the polarization direction of the excitation light can be arranged to be parallel to the emission path. In this case the detector, measures signals ${I}_{\perp }$ and ${I}_{\perp }^{* }$, which are both polarized at right angles to the polarization state of the excitation light [1], regardless of the analyzer orientation. Note that spectrofluorometers typically have only one detector, while the analyzer is rotated. This has the benefit of equal background noise levels for both polarization directions. Hence, both detectors should record the same amount of fluorescence signal. This holds true as long as RD of the fluorophore is rotationally symmetric around the axis of the excitation polarization. Any deviation from unity in the *G* factor must therefore be due to a polarization dependence of the imaging system, which can be expressed as

2 \begin{eqnarray*}{G}_{\mathrm{spectrofluorometer}}=\displaystyle \frac{{I}_{\perp }}{{I}_{\perp }^{* }}.\end{eqnarray*}

**Figure 1.**
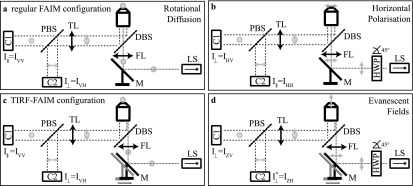
(a) Sketch of a typical FAIM setup, representative of the one used in this study. The system contains a polarized light source (LS), which illuminates the sample through the objective lens in an inverted microscope configuration. The emitted fluorescence is captured by the same objective and coupled out by a dichroic beam splitter (DBS) and directed towards the tube lens (TL). A polarizing beam splitter (PBS) then transmits one polarization onto the first camera (*C*1) and the orthogonal polarization onto the second camera (*C*2). No changes have to be made to this experimental configuration to apply the protocol for *G* factor determination via the RD method. (b) For the horizontal polarization, HP, calibration method, an additional measurement has to be taken, which requires the excitation light to be polarized orthogonally to the primary excitation field and the optical axis. This can be achieved via a half wave plate (HWP) in the excitation beam path, oriented with its fast transmission axis at ${45}^{\circ }$ to the polarization of the excitation light. The HP method is considered to be the ‘gold standard’ for *G* factor calibration. (c) For FAIM with TIR illumination the excitation light is focused by the focusing lens (FL) into the TIR ring in the back focal plane of the objective lens rather than the center. In the depicted scheme this is achieved by translating mirror M. Although this ensures a great increase in contrast, the HP method can no longer be employed for *G* factor calibration, because the two orthogonal polarization states cannot be produced in the excitation light. (d) Instead, the evanescent field, EF, method can be used, which we propose here: a horizontal polarization in the excitation beam leads to an excitation of the sample along a direction orthogonal to both detection arms (see figure 2), and this can be used for *G* factor calibration analogously to what is traditionally done for spectrofluorometers (see equation (2)).

In an inverted fluorescence microscope this approach is not possible as the path for excitation of the fluorophores is co-axial with, rather than perpendicular to, the detection path. Hence, alternative approaches are required that are suitable for calibrating co-axial imaging systems, of which an example is depicted in figure 1(a). One such approach for calibration makes use of rapidly rotating small fluorescent dye molecules, which feature a long fluorescent lifetime and are contained in a low viscosity solution [4]. In what follows, we will refer to this method via the subscript RD for *rotational diffusion*.

Fluorophores with a short fluorescence lifetime hardly alter their dipole orientation in space between excitation and emission events and thus the resulting emission light remains highly polarized. Molecules with a long fluorescence lifetime, on the other hand, stay in their excited states long enough for considerable rotation of the molecules to occur. Consequently, the axes of the emission dipoles are also rotated into random positions and the fluorescence emitted from the ensemble of fluorophores becomes increasingly isotropic in time. Small molecules in low viscosity environments feature fast RD, and thus their emission polarization becomes fully isotropic over a period of the fluorescence lifetime. Thus, any residual anisotropy detected for the latter sample is due to polarizing components in the optical setup and the fluorophore is a suitable calibration standard. A commonly used reference sample with suitable properties is a dilute solution of fluorescein in water ($\lt 0.1\,\mu {\rm{M}}$). The RD method appeals due to its simplicity but comes with a number of drawbacks. Firstly, due to the wavelength dependence of the *G* factor, a calibration fluorophore should be used that is spectrally matched to the fluorescent molecule under investigation and that also exhibits a long emission lifetime and a fast RD coefficient (small hydrodynamic radius). Slowly rotating reference fluorophores can however also be used for reference if their anisotropy is determined in a well-calibrated spectrofluorometer for the same solution conditions as subsequently used for calibration of the FAIM microscope. The microscope *G* factor can in this case be calculated using the known anisotropy *r*_ref_ of the reference fluorophore:

3 \begin{eqnarray*}{G}_{\mathrm{RD}}=\displaystyle \frac{{I}_{\parallel }(1-{r}_{\mathrm{ref}})}{{I}_{\perp }(1+2{r}_{\mathrm{ref}})}=\displaystyle \frac{{I}_{\parallel }^{0}}{{I}_{\perp }^{0}}=\displaystyle \frac{{I}_{{VV}}}{{I}_{{VH}}}=\displaystyle \frac{I(C1)}{I(C2)}.\end{eqnarray*}

Here *I_VV_* and *I_VH_* are the images taken by cameras *C*1 and *C*2 for vertical excitation polarization as depicted in figure 1(a). Subscripts *V* and *H* refer to vertical and horizontal polarizations (HPs), which we define with reference to the optical table surface. For example, *I_VH_* refers to excitation light with the polarization direction perpendicular to the optical table on exit from the excitation source, and with the signal parallel to the table surface at the detector; see figure 1. Note that the second equality in equation (3) holds for rapidly rotating dyes, for example dilute fluorescein solutions, which feature ${r}_{\mathrm{ref}}\approx 0$. An alternative way to estimate the *G *factor is by rotating the excitation polarization [5, 6]. As seen in figure 1(b), a half wave plate (HWP) arranged with its fast axis at ${45}^{\circ }$ to the laser polarization direction results in light polarized horizontally. Together with the configuration in figure 1(a) this enables the recording of a set of four measurements $[{I}_{{HH}},{I}_{{HV}},{I}_{{VH}},{I}_{{VV}}]$, from which one can calculate *G* via

4 \begin{eqnarray*}{G}_{{\rm{HP}}}=\sqrt{\displaystyle \frac{{I}_{{VV}}}{{I}_{{VH}}}\displaystyle \frac{{I}_{{HV}}}{{I}_{{HH}}}}=\sqrt{\displaystyle \frac{{I}_{{V}}(C2)}{{I}_{{V}}(C1)}\displaystyle \frac{{I}_{{H}}(C2)}{{I}_{{H}}(C1)}}.\end{eqnarray*}

**Figure 2.**
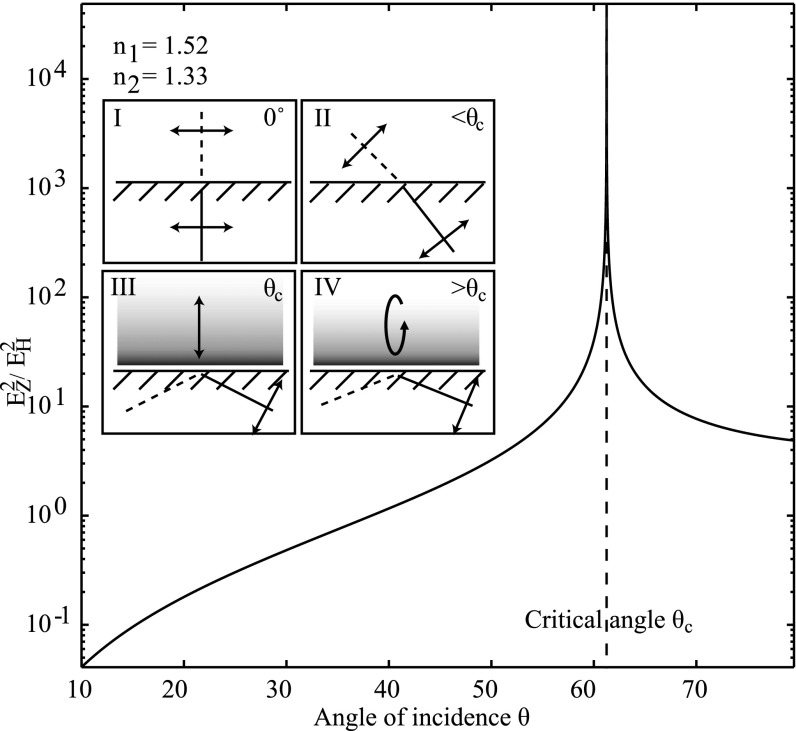
Polarization state of excitation light in the focal plane of the objective lens as a function of incidence angle. The inlays I–IV show how incident p-polarized light is bent due to refraction on the coverslip surface until it reaches the critical angle ${\theta }_{{c}}$, where total internal reflection illumination results. This evanescent field is polarized perpendicular to the coverslip surface. At inclinations beyond the critical angle the polarization of the evanescent field becomes elliptical.

We will refer to this approach with the subscript HP for *horizontal polarization*. Although HP is the gold standard method for *G* factor calibration in FAIM experiments, it cannot be applied to microscopes, in which the sample is excited with oblique (HILO) [7] or TIRF modes of illumination. This is because the illumination in HILO and TIRF is tilted with respect to the optical axis of the objective lens, and thus it is not possible to arrange for the excitation light to be polarized parallel to one of the detection arms. The four panels in figure 2 illustrate this.

In panel (I), the illumination light is focused into the center of the back focal plane of the objective, hence the direction of the emerging light on the sample-facing side is along the optical axis, as defined by the objective, and the polarization is hence perpendicular to it. In panel (II), the excitation light is focused off-center in the back focal plane of the objective causing the illumination beam to leave the objective under an angle $\theta $. Refraction on the coverslip-sample interface further tilts the direction of the excitation light according to Snell's law. For the polarization direction of the transmitted light two cases have to be distinguished. The s-polarization of the light (corresponding to figure 1(b)) is independent of $\theta $, but the p-polarization is tilted along with the beam. After passing through the coverslip, the resulting polarization can be described as the ratio between the portion of light polarized in *z* (along the optical axis of the objective) and that in the direction of *on-axis* p-polarized light, i.e. the HP as sketched in figure 1. According to [8], the polarization state is then

5 \begin{eqnarray*}\displaystyle \frac{{I}_{Z}}{{I}_{H}}=\displaystyle \frac{\left|{\left(\tfrac{\sqrt{{n}_{2}^{2}-{n}_{1}^{2}{\sin }^{2}(\theta )}{t}_{\mathrm{TM}}(\theta )}{{n}_{2}}\right)}^{2}\right|}{\left|{\left(\tfrac{{n}_{1}{\sin }^{2}(\theta ){t}_{\mathrm{TM}}(\theta )}{{n}_{2}}\right)}^{2}\right|}\end{eqnarray*}

with

6 \begin{eqnarray*}{t}_{\mathrm{TM}}(\theta )=\displaystyle \frac{2{n}_{1}{n}_{2}\cos (\theta )}{{n}_{2}^{2}\cos (\theta )+{n}_{1}\sqrt{{n}_{2}^{2}-{n}_{1}^{2}{\sin }^{2}(\theta )}}.\end{eqnarray*}

A graph of equation (5) is plotted in figure 2. *I*_*Z*_ and *I*_*H*_ represent the components along the optical axis and parallel to the coverslip surface in the plane spanned by the incoming and refracted beams. *n*_1_ and *n*_2_ are the refractive indices of coverslip and sample medium. Once the inclination of the beam reaches the critical angle ${\theta }_{{c}}$ as depicted in panel (III) of figure 2, the light produces an EF, which is polarized in a perpendicular direction to the surface and thus the graph of equation (5) approaches infinity as $\theta $ approaches ${\theta }_{{c}}$. Incident light above the critical angle causes the EF to be elliptically polarized (panel (IV)). It can be seen that, as soon as $\theta \ne 0^\circ $, equation (4) cannot strictly be applied anymore, as the illumination is no longer purely horizontally polarized. Although a strongly inclined excitation prevents the use of the HP method, the polarization of the EF at the critical angle is orthogonal to both camera directions (see graph in figure 2). Hence, we propose here that it is possible to combine the collinear geometry of an anisotropy TIRF microscope with the precise *G* factor calibration method that is conventionally used for an L-format spectrofluorometer (see figure 1(d)). Due to the geometric similarity, the formula given in equation (2) for a spectrofluorometer can also be applied to calculate the *G* factor in a TIRF-FAIM microscope:

7 \begin{eqnarray*}{G}_{\mathrm{EF}}=\displaystyle \frac{{I}_{\perp }^{* }}{{I}_{\perp }}=\displaystyle \frac{{I}_{{ZV}}}{{I}_{{ZH}}}=\displaystyle \frac{I(C2)}{I(C1)}.\end{eqnarray*}

Here the subscript EF abbreviates the proposed *evanescent field* calibration method and the subscript *Z* indicates excitation along the optical axis of the objective lens, which occurs at the critical angle upon internal reflection of highly inclined excitation light. In what follows we implement the method experimentally and prove its validity.

## 2. Results and discussion

As sketched in figures 2(c) and (d), the FAIM set-up used for this work contained a HWP (WPH05M-488, Thorlabs) placed after a vertically polarized 488 nm laser line (Coherent Sapphire) and a mirror mounted on a translation stage (BB2-E02 on NRT100/M, Thorlabs) to accurately and reproducibly switch between episcopic and TIRF illumination modes. The focusing lens (AC508-400-A, Thorlabs) was also mounted on the translation stage. A commercial microscope frame (Olympus IX73) with a 100×, NA = 1.49 oil immersion TIRF objective (Olympus UAPON100XOTIRF) was used and fluorescence coupled out by a dichroic beam splitter (Chroma ZT405/488/561/640rpc) used in combination with a 525/45 emission filter (Semrock). A polarizing beam splitter (PBS) was mounted in a dual camera splitter (TwinCam, CAIRN), which contained an additional clean-up polarizer for the reflected light (both PBS and clean-up polarizer from CAIRN). Two identical EMCCD cameras (iXon Ultra 897, Andor) were used for detection, both of which had a measured effective pixelsize of 118 nm, 512 × 512 pixels, and less than 1e^−^ read noise. Before anisotropy imaging can be performed, several calibration steps have to be performed. These include, apart from the *G* factor determination, a quantification of the polarization leakage arising from imperfections of the PBS, and depolarization and polarization mixing caused by the large acceptance angle of the high numerical aperture objective used [9]. Note that the *G* factor also accounts for different camera exposure times, which are an obvious source of bias in the determination of anisotropy values. This is especially important as in the presented set-up camera 1 triggered camera 2, which resulted in slightly different exposure times of 0.1 and 0.08 s. Background images were taken separately for both cameras and acquisition settings. These factors are considered in detail in the following paragraphs.

### 2.1. Depolarization effects caused by high NA objectives

To quantify the depolarization caused by the objective acceptance angle, we used a $0.1\,\mu {\rm{M}}$ solution of fluorescein in 40% glycerol. We chose fluorescein in 40% glycerol to provide a reference anisotropy value, which is close to that observed in cells. We imaged with three different objectives; a 1.49 NA objective (Olympus UAPON100XOTIRF), a 0.7 NA objective (Olympus UCPlanFL N), and a 0.04 NA objective (Olympus PLAPON). This allowed us to perform a calibration for the effect of depolarization by high NA lenses using the method previously described in [5, 10]. Large collection angles of high NA objectives result in mixing of different polarization states and hence a reduction in the measured anisotropy values. Smaller NAs of the objective lens cause smaller mixing effects and thus measurements more closely approximate the unperturbed values, e.g. those that would be determined by a spectrofluorometer. We compared two methods, which are commonly used to correct for the influence of high NA optics. The first method follows the theoretical model presented by Axelrod [12], which can be used to calculate correction factors for the measured ${I}_{\perp }$ and ${I}_{\parallel }$ with respect to the real values *I*_*x*_ and *I*_*y*_ upon illumination with light in the *y*-polarized direction. This method is especially appealing as no calibration is neccessary:

8 \begin{eqnarray*}\left[\begin{array}{c}{I}_{\perp }\\ {I}_{\parallel }\end{array}\right]=\left[\begin{array}{cc}{K}_{a}+{K}_{c} &amp; {K}_{b}\\ {K}_{a}+{K}_{b} &amp; {K}_{c}\end{array}\right]\left[\begin{array}{c}{I}_{x}\\ {I}_{y}\end{array}\right],\end{eqnarray*}

where

9 \begin{eqnarray*}{K}_{a} &amp; = &amp; 1/3(2-3\cos (\alpha )+{\cos }^{3}(\alpha )),\\ {K}_{b} &amp; = &amp; 1/12(1-3\cos (\alpha )+3{\cos }^{2}(\alpha )-{\cos }^{3}(\alpha )),\\ {K}_{c} &amp; = &amp; 1/4(5-3\cos (\alpha )-{\cos }^{2}(\alpha )-{\cos }^{3}(\alpha )).\end{eqnarray*}

Without any polarization mixing ${I}_{\perp }={I}_{x}$ and ${I}_{\parallel }={I}_{y}$. Note that this equation assumes ${I}_{x}={I}_{z}$, which is valid for randomly oriented systems (*z* is along the optical axis). Solving equation (8) for *I*_*x*_ and *I*_*y*_ yields

10 \begin{eqnarray*}\left[\begin{array}{c}{I}_{\perp }^{\mathrm{corrected}}\\ {I}_{\parallel }^{\mathrm{corrected}}\end{array}\right]\,=\,\left[\begin{array}{c}{I}_{x}\\ {I}_{y}\end{array}\right]\,=\,\displaystyle \frac{1}{({K}_{b}-{K}_{c})({K}_{a}+{K}_{b}+{K}_{c})}\\ \quad \times \,\left[\begin{array}{cc}-{K}_{c} &amp; {K}_{b}\\ {K}_{a}+{K}_{b} &amp; -{Ka}-{K}_{c}\end{array}\right]\left[\begin{array}{c}{I}_{\perp }\\ {I}_{\parallel }\end{array}\right].\end{eqnarray*}

**Figure 3.**
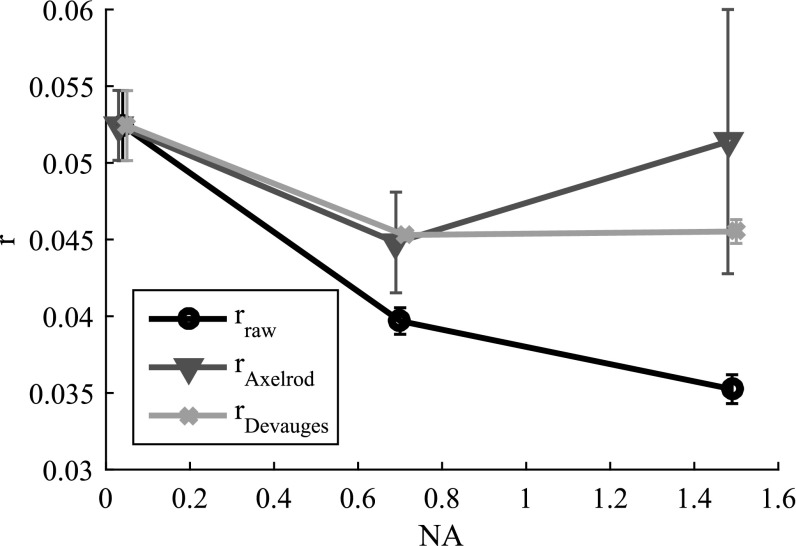
The influence of polarization mixing in high NA lenses leads to decreased measured anisotropy values. The effect was assessed using a fluorescein containing viscous glycerol solution. The measured anisotropy is seen to decrease dramatically with increasing NA from the true value of of $r=0.053$. Both correction methods produced similar results for an intermediate NA of 0.7, whereas for high NAs, the theoretical method yields a result closer to the ground truth as determined by the low NA objective. Despite slightly less correction quality of the calibration-based Devauges method, the correction values are more reproducible than the ones predicted by the theory-based Axelrod method in the presence of noise and other polarization-mixing effects. Measurement points are the means and error bars of the standard deviations of 6 measurements.

We find that this method achieves satisfactory correction results for our measurements, but also that the measurements are sensitive to noise. Furthermore, the Axelrod method only accounts for the polarization mixing effect caused by the objective lens but leaves out all other possible influences, which might be the reason for the large standard deviations observed in figure 3. An alternative method, presented by Devauges *et al* [10], determines the real anisotropy values from measurements using a high NA objective by reference to anisotropy values determined by a low NA objective and application of a correction factor *x*_NA_:

11 \begin{eqnarray*}{r}_{\mathrm{Devauges}}=\displaystyle \frac{{I}_{\parallel }-{{GI}}_{\perp }}{{I}_{\parallel }+{x}_{\mathrm{NA}}{{GI}}_{\perp }}.\end{eqnarray*}

The correction factor *x*_NA_ is calculated as

12 \begin{eqnarray*}{x}_{\mathrm{NA}}=\displaystyle \frac{{I}_{\parallel }^{h}(1-{r}^{l})-{G}^{h}{I}_{\perp }^{h}}{{G}^{h}{r}^{l}{I}_{\perp }^{h}}.\end{eqnarray*}

The superscripts *h* and *l* indicate measurements performed with high and low numerical aperture objectives, respectively. Further information can also be found in [11]. The results of both methods to correct anisotropy values for the different objectives are shown in figure 3.

The correction factors produced by the Devauges method are more stable, as indicated by the smaller standard deviation of repeated measurements, but they underestimate the extent of correction needed compared to the method proposed by Axelrod. We conclude that for systems with other uncorrected sources of depolarization and polarization mixing, not just those due to high NA objectives, the use of the calibration based Devauges method is preferable.

### 2.2. Correction of polarization leakage

We also estimated the leakage of vertical polarization into the HP path, and vice versa, arising from imperfections of the PBS. For this we used brightfield illumination with perfectly polarized light produced by a Glan–Taylor polarizer (GL10, Thorlabs) placed under the brightfield lamp, which featured an extinction ratio of 10^6^:1, and imaged the surface of a coverslip onto the two cameras after the PBS. The polarization leakage in our system was found to be neglible (approx. 0.6%) but can be much higher in general (i.e. a value of 10% was measured by [6] in their system). Note that unpolarized brightfield illumination can not be used for *G* factor determination in general as the lamp's spectrum is ususally very different to fluorophore emission spectra.

### 2.3. *G* factor determination

**Figure 4.**
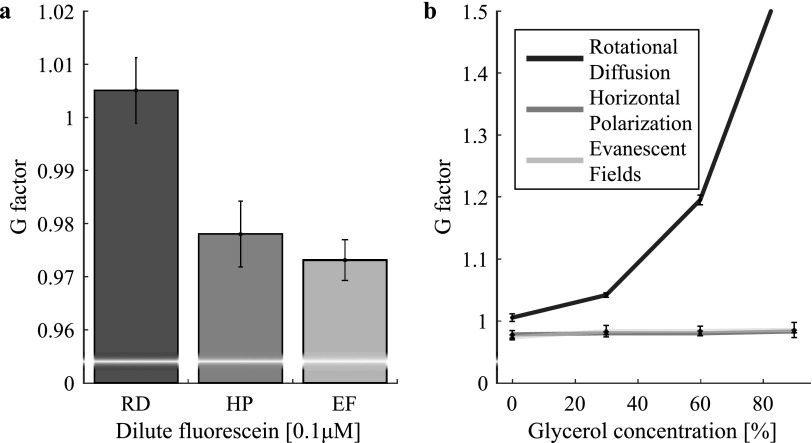
(a) The* G* factor was calculated using the three presented methods and found to be close to 1 in all three cases as expected for a low viscosity solution of fluorescein. The value produced by the RD method was higher than the value measured with the other two methods, which indicates inaccuracies in the reference anisotropy *r*_ref_ used. (b) This effect becomes clearer when increasing the amount of glycerol in the solution so as to increase viscosity and rotational diffusion times. One sees clearly how the RD method starts to deviate dramatically for higher viscosities (no correction for *r*_ref_ was applied). The HP and EF methods provide stable measurements of the *G* factor. Measurement points are the means of 5 measurements and error bars represent the standard deviations.

The *G* factor of our system was first determined by the established RD and HP methods and was then used to validate the proposed EF method. In particular, we prepared and imaged multiple samples containing $0.1\,\mu {\rm{M}}$ of fluorescein in water with increasing proportions of glycerol. Prior to anisotropy imaging a bead sample was recorded with both cameras. Using descriptor-based registration between the two bead images [13] all FAIM raw data was aligned before any other inter-image processing was performed. We took five independent measurements of each glycerol condition and for each state of excitation polarization (horizontal, vertical, axial). This was achieved in our set-up by ${45}^{\circ }$ rotation of the HWP to change between vertical and horizontal illumination and translation of the mirror to focus horizontal excitation light into the TIR ring of the objective to allow for critical illumination and hence axial polarization. As a metric of comparison for the EF method with respect to the RD and HP methods, we computed *G* factors for the central 256 × 256 pixels of the aligned raw images. The mean was used as the *G* factor determined for a given glycerol concentration and method. The measurement points are displayed in figure 4 and represent the mean of 5 such measurements. Error bars indicate the standard deviation.

The *G* factor is, as expected, found to be close to 1 for all three methods. Note that the EF method yields results that are insignificantly different to the gold standard HP method ($p=0.22$ with Mann–Whitney test), whereas a slight difference is present with respect to the RD method ($p=0.008$) as is displayed in figure 4(a). This error is likely due to a slightly incorrect reference anisotropy *r*_ref_, which is required to compute *G*_RD_ accurately. The effect of slowed RD becomes even more prominent with increasing viscosity of the sample, which is plotted in figure 4(b). Here, the RD method produces vastly divergent results if *r*_ref_ is not corrected for, whereas EF and HP offer stable and almost identical results. Note that misalignment and deviations from critical angle excitation would cause a slight deterioration of the EF method as well, albeit to a much lesser extent than the one observed with the RD method. This can be attributed to two effects. Firstly, portions of vertical illumination are generated from s-polarized light stemming from misalignment and, secondly, due to a HP component generated from p-polarized illumination at non critical angles. Both of these polarization states are mixed with the wanted axial polarization. As the measured anisotropy signal under vertical and horizontal illumination is affected at higher viscosities, a similar dependence as observed in the RD method would be the result. In practice, focusing the excitation light at the edge of the TIR ring of the objective's back focal plane is crucial but readily achievable in an optical set-up as described in this article. Proper alignement can be checked when using two samples of dilute fluorescein—one in water and one in glycerol. Under optimal conditions, *G* factor measurements of both samples will yield the same result. Also note that functionalized surfaces might cause preferential alignment of fluorophores, which might hinder isotropic emission despite axial illumination polarization. This is especially important for the EF method as only a thin layer near the coverslip is investigated.

### 2.4. Anisotropy and protein polymerization measurements in cells

After system calibration, we imaged different cell types to study actin polymerization in live cells. In previous studies [14] we had discovered a great impact of the actin production on the development of neuronal connections. In particular, we found that the monomeric protein $\beta $-actin, the building block of the polymerized fibrilar F-actin, is expressed with great spatial variance. To investigate this further, we used our TIRF-FAIM microscope to visualize the polymerization state of actin in different cell types. First, we imaged human embryonic kidney cells (HEK293T), which were transfected with a construct consisting of the yellow fluorescent protein *Venus* and the coding sequence for $\beta $-actin (labeled via DNA transfection as described elsewhere [14]) and cultured in MatTek 35 mm dishes for imaging. The HEK293T cells were used to assess the effects of TIRF and episcopic illumination respectively on the sensitivity and quality of anisotropy measurements and to image the distribution of F-actin with respect to $\beta $-actin in this cell type. The intensity-weighted polymer fraction *C*_polymer_ can be calculated from the anisotropy [2], with *r* being the measured anisotropy, *I* the total intensity and *r*_*m*_ the fluorescence anisotropy from individual monomers:

13 \begin{eqnarray*}{C}_{{\rm{polymer}}}\propto \left(1-\displaystyle \frac{r}{{r}_{m}}\right)I.\end{eqnarray*}

**Figure 5.**
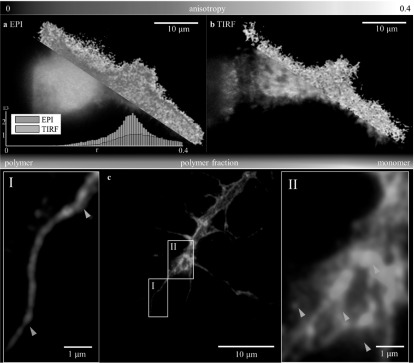
(a) Anisotropy and polymerization measurements in HEK293T cells show out-of-focus haze in and around the cell, which is clearly reduced when switching from EPI to TIRF illumination as shown in (b). Similarly, the large fraction of $\beta $-actin polymer in cell filopodia is much stronger in contrast against the background. (c) The same Venus-$\beta $-actin construct was used to label retinal neurons cultured from eye primordia. (I) Shows almost pure monomeric $\beta $-actin at the base of the filopodium (orange arrowhead), while the tip is comprised of mostly polymerized F-actin (blue arrowhead). The brightness of panel (I) was increased by 50% for better visibility. Panel (II) shows that the center of the growth cone is a mixture of polymeric and monomeric actin but with clearly distinct monomeric spots (orange arrowheads). These spots could stem from spatially distinct translation sites [14], which locally increase the portion of monomeric $\beta $-actin. The inlay shows a histogram comparing the anisotropy values measured under episcopic and TIRF illumination.

Under episcopic (EPI) illumination, see figure 5(a), the imaged HEK293T cells were bathed in a strong haze of out-out-focus light and this prohibited anisotropy measurements to be performed quantitatively so that the $\beta $-actin polymerization process could be followed. Avoiding out-of-focus light through TIRF illumination led to a much more detailed view of anisotropy variations within the cell (figure 5(b)). Hereby, we used s-polarized light under critical illumination.

In particular, the calculated polymer-fraction maps show a clear increase in polymerized $\beta $-actin in the filopodia of the cells, which is consistent with previous measurements using two-photon FAIM [2]. As both polarization components that are necessary for anisotropy calculation are recorded simultaneously, it is also possible to record high framerate movies (see supplementary video S1 available online at stacks.iop.org/MAF/6/014004/mmedia). Next, the same Venus-$\beta $-actin construct was expressed in retinal neurons, which allowed us to study the spatial variance of polymerization during neuronal growth and potential deviations between the different cell types. Figure 5(c) shows a growth cone, the end-tip of a growing axon, of a retinal neuron during the axonal navigation stage. Similarly to the HEK293T cells, a larger fraction of polymeric $\beta $-actin can be found in the filopodia of the cells (see panel (I) for an enlarged view). Zooming into the core of the growth cone (panel (II)), we indeed find faint puncta of enhanced monomer fraction compared to the surrounding axoplasm (orange arrow heads point to example puncta). We note that these seemingly monomeric puncta might not be due to enhanced expression or accumulation of protein but could be artefacts caused by substrate interactions with the cell, resulting in enhanced autofluorescence of debris, which can appear punctate. Further investigations are necessary in this respect to correlate the observed monomeric puncta to biological function and rule out systematic errors in the culturing and labeling process.

## 3. Conclusion

In summary, we have developed a new method for calibration of a TIRF-FAIM microsope, making use of excitation light, which is polarized orthogonal to both detection polarizations. We achieved this by illumination of the calibration sample with p-polarized light incident at the critical angle, so that total internal reflection results in an EF polarized along the optical axis. We compared our method against two established methods for *G* factor calibration and verified the validity and practical advantages of our approach. Our measurements suggest, that the EFs method proposed here has similar performance as the gold standard method, which requires double excitation under crossed polarizations. Also, other calibration steps were investigated to account for high NA depolarization effects, which are particularly important in TIRF microscopy setups. In particular, we compared the calibration-free correction based on theoretical predictions by Axelrod [12] to the calibration method introduced by Devauges [10], which makes use of a second, low NA objective. We report that both methods achieve comparable results for modest NAs. At high NAs, however, Devauges calibration method appears to be more reliable as depolarization effects other than those caused by the high NA can be accounted for. With our calibrated TIRF-FAIM setup using s-polarized excitation we were able to record movies of migrating HEK293T cells displaying higher levels of polymerized $\beta $-actin in their filopodia. Furthermore, we imaged the spatial distribution of polymer-to-monomer fraction in cultured neuronal growth cones during axonal navigation. We find evidence to suggest local translation takes place in growth cones, distal to the cell body [14] and that this results in increased local monomer densities. In addition, we have developed a comprehensive software package called *AniCalc* to process polarization data into anisotropy images and make it freely available at *laser.ceb.cam.ac.uk* as a plug-in for ImageJ/Fiji [15]. All raw data and software used to process the anisotropy images into polymerization maps are available from FS on request.

## 

## References

[1] Lakowicz J R (2006). Principles of Fluorescence Spectroscopy.

[2] Vishwasrao H D, Trifilieff P, Kandel E R (2012). *In vivo* imaging of the actin polymerization state with two-photon fluorescence anisotropy. Biophys. J..

[3] Davies T, Kodera N, Kaminski-Schierle G S, Rees E, Erdelyi M, Kaminski C F, Ando T, Mishima M (2015). CYK4 promotes antiparallel microtubule bundling by optimizing MKLP1 neck conformation. PLoS Biol..

[4] Chan F T S, Kaminski C F, Kaminski-Schierle G S (2011). HomoFRET fluorescence anisotropy imaging as a tool to study molecular self-assembly in live cells. ChemPhysChem.

[5] Tramier M, Kemnitz K, Durieux C, Coppey J, Denjean P, Pansu R B, Coppey-Moisan M (2000). Restrained torsional dynamics of nuclear DNA in living proliferative mammalian cells. Biophys. J..

[6] Siegel J, Suhling K, Leveque-Fort S, Webb S E D, Davis D M, Phillips D, Sabharwal Y, French P M W (2003). Wide-field time-resolved fluorescence anisotropy imaging (TR-FAIM): imaging the rotational mobility of a fluorophore. Rev. Sci. Instrum..

[7] Tokunaga M, Imamoto N, Sakata-Sogawa K (2008). Highly inclined thin illumination enables clear single-molecule imaging in cells. Nat. Methods.

[8] Józefowski L, Fiutowski J, Kawalec T, Rubahn H G (2007). Direct measurement of the evanescent-wave polarization state. J. Opt. Soc. Am. B.

[9] Erdelyi M, Simon J, Barnard E A, Kaminski C F (2014). Analyzing receptor assemblies in the cell membrane using fluorescence anisotropy imaging with TIRF microscopy. PLoS One.

[10] Devauges V, Marquer C, Lécart S, Cossec J C, Potier M C, Fort E, Suhling K, Lévêque-Fort S (2012). Homodimerization of amyloid precursor protein at the plasma membrane: a homoFRET study by time-resolved fluorescence anisotropy imaging. PLoS One.

[11] Suhling K, Levitt J, Chung P H (2014). Time-resolved fluorescence anisotropy imaging. Fluorescence Spectroscopy and Microscopy: Methods and Protocols.

[12] Axelrod D (1979). Carbocyanine dye orientation in red cell membrane studied by microscopic fluorescence polarization. Biophys. J..

[13] Preibisch S, Saalfeld S, Schindelin J, Tomancak P (2010). Software for bead-based registration of selective plane illumination microscopy data. Nat. Methods.

[14] Ströhl F, Lin J Q, Laine R F, Wong H H W, Urbančič V, Cagnetta R, Holt C E, Kaminski C F (2017). Single molecule translation imaging visualizes the dynamics of local *β*-actin synthesis in retinal axons. Sci. Rep..

[15] Schindelin J (2012). Fiji: an open-source platform for biological-image analysis. Nat. Methods.

